# Elimination of tropical disease through surveillance and response

**DOI:** 10.1186/2049-9957-2-1

**Published:** 2013-01-03

**Authors:** Xiao-Nong Zhou, Robert Bergquist, Marcel Tanner

**Affiliations:** 1 National Institute of Parasitic Diseases, Chinese Center for Disease Control and Prevention, Shanghai, 200025, People’s Republic of China; 2 WHO Collaborating Centre for Malaria, Schistosomiasis and Filariasis, Key Laboratory of Parasite and Vector Biology, Ministry of Health, Shanghai, 200025, People’s Republic of China; 3 Ingerod, 407, Brastad, Sweden; 4 Department of Epidemiology and Public Health, Swiss Tropical and Public Health Institute, P.O. Box, Basel, CH-4002, Switzerland; 5 University of Basel, P.O. Box, Basel, CH-4003, Switzerland

## Abstract

Surveillance and response represent the final crucial steps in achieving effective control and particularly elimination of communicable diseases as recognized in the area of neglected tropical diseases (NTDs), applied in increasing numbers in endemic countries with ongoing control and elimination programmers. More and more national NTD elimination initiatives are scheduled based on the innovative and effective One world-One health perspective to detect pockets of transmission and disease reintroduction. Resource-constrained countries, which carry the heaviest NTD burdens, face various challenges how to strengthen the health system as well as developing effective and novel tools for surveillance and response tailored to local settings. Surveillance-response approaches take place in two different stages corralling the basic components of the surveillance-response system for NTD elimination. Six different research priorities have been identified:1)dynamic mapping of transmission, 2) near real-time capture of population dynamics, 3) modelling based on a minimum essential database/dataset, 4) implementation of mobile health (m-health) and sensitive diagnostics, 5) design of effective response packages tailored to different transmission settings and levels, and 6) validation of approaches and responses packages.

## Background

In 2011, the World Health Organization (WHO) Strategic and Technical Advisory Group for Neglected Tropical Diseases (NTDs) and its partners among other influential organizations, adopted a roadmap for control, elimination and eradication [1]. Inspired by this roadmap, global health leaders, including the Chief Executive Officers (CEOs) of major pharmaceutical companies, Bill Gates of the Bill & Melinda Gates Foundation, WHO Director General Margaret Chan and senior government officials from both endemic and donor countries, signed the London Declaration, which represents an unprecedented commitment to control or eliminate 10 diseases by the end of this decade [2]. All stakeholders believe in charting a new course toward health and sustainability among the world’s poorest communities offering them a stronger, healthier future through implementation of the declaration. The goal is to eradicate blinding trachoma, leprosy, human African trypanosomiasis, lymphatic filariasis globally by 2020, while rabies, endemic treponematoses (yaws), Chagas disease, visceral leishmaniasis, onchocerciaisis and schistosomiasis are targeted for elimination at the regional level at the same time [1,3]. More recently, a growing number of countries has adopted malaria elimination as a goal in response to the global malaria elimination programme [4]. For example, the African Union’s 2007 “Africa Malaria Elimination Campaign”, the recent declaration by the Bill and Melinda Gates Foundation of reinstating global eradication as a long-term objective, and the reiterated support by the Director General of WHO, have all reinforced the goal of local elimination, as well as global eradication not only as a vision, but also as a realistic, long-term objective [5,6]. Success would represent one of the most cost-effective means to lift more than 1 billion people out of poverty and prevent needless suffering among future generations [3,4].

Elimination of a disease is defined as reducing a locally acquired infection to zero incidence in a specific, geographic area through deliberate efforts, leading to interruption of its transmission [7,8]. To achieve this, it is necessary to formulate a strategy for elimination and prevention of disease reintroduction at the national level. Strengthening the health system is particularly important for the national elimination programme in the developing countries [9]. Technically, higher coverage of intervention is encouraged to sustain and maintain what has already been achieved and this can only be done by a highly efficient programme management. As has been experienced by the polio and smallpox eradication programmes [10,11], surveillance and response systems appear to be the most cost-effective way to improve the efficiency in disease elimination. Intervention needs to target the heterogeneity of transmission, especially with respect to the identification and rapid elimination of foci of all infections, both symptomatic and asymptomatic [12]. However, the concept and role of a surveillance and response system for disease elimination have not been fully developed and validated for all different types of disease and all different epidemiological settings [13]. Questions surrounding these areas were discussed during The First Forum on Surveillance Response System Leading to Tropical Diseases Elimination, held in Shanghai, Peoples’ Republic of China (P.R. China) on 16–17 June, 2012, with the aim to explore novel approaches towards the establishment of integrated surveillance-response systems that would enable disease elimination efforts.

## Surveillance and response systems under the health systems framework

The concept of surveillance and response evolved from the original vision of the general and open-ended-term “surveillance”, which can now be defined as the ongoing systematic collection, analysis, and interpretation of health data [14]. It is aimed at discovery, investigation, and elimination of continuing transmission, the prevention and cure of infection and final substantiation of claimed eradication [14,15]. Timely dissemination of surveillance results can improve planning, implementation, and evaluation of public health practice, by using at least four different approaches, i.e. health facility-based or community-based surveillance, sentinel surveillance, laboratory-based surveillance, and disease-specific surveillance [16]. For example, an effective schistosomiasis surveillance system enables programme managers to identify the risk areas, or the population groups most affected, identify trends in both human and animal cases that require intervention, and assess the efficiency and impact of control measures [17,18].

The surveillance and response approach entails the One world-One health perspective geared at detection, reporting, analysis, interpretation and action for public health by integrating and streamlining common surveillance activities [19]. However, to comply with the overriding aim of disease elimination, it should be followed by an effective public health response – delivered as integrated packages – with the purpose to interrupt transmission in well defined areas. The other key feature is that surveillance and response systems are based on a set of minimum essential data aiming at the capture of foci/pockets of transmission or disease reintroduction. This approach is different from the classical monitoring and evaluation with its focus on collecting all possible data, which often leads to information overflow as well as lack of feedback and rapid effective public health action. The One world-One health perspective also contains the strategy that addresses events at the intersection of human, domestic animal, wildlife, and ecosystem health situation [20]. Its effective and timely public health responses depend upon the ability of health systems to provide accurate and timely information for action [21]. The global smallpox and polio eradication programmes provide excellent examples of the critical role that surveillance plays in linking surveillance data to targeted public health responses [10,11]. The desired performance of the surveillance and response systems is to generate information for timely action contributing to the reduction of mortality, disability and morbidity for the targeted diseases, e.g. epidemic-prone diseases, and diseases targeted for eradication and elimination [16]. However, in many resource constrained countries, health systems and thus surveillance systems provide a weak response capacity to emerging threats due to scarce resources, except for selected high-priority diseases [22,23]. As shown here, the One world-One health approach is able to overcome two of the abovementioned problems in health systems research and the application of an integrated strategy [20,24].

The first issue of the Infectious Diseases of Poverty journal, with its theme of health system for infectious diseases of poverty, called for countries with strong surveillance systems to facilitate their national elimination programmes by improving their health systems with respect to detection, notification and launching of public health responses that can manage foci of transmission as well as outbreaks/epidemics, and individual cases [25,26]. Such systems are an integral part of health systems with the structural (facilities, equipment) and functional (mainly human resources) capacities. For example, with regard to malaria elimination, in-door spraying in transmission foci is one of the response measures that needs to be performed immediately once such foci are found. However, these actions need to be complemented by active case detection and treatment among all inhabitants and migrants in the suspected remaining pocket of transmission [25,27].

## The role of surveillance and response in disease elimination

Countries do generally not attempt to initiate elimination efforts for any disease until an intensive surveillance system is in place. This fact not only emphasizes the need to develop effective surveillance systems based on the minimum essential data concept, but also serves to design public health response packages for the different endemic settings. Clearly, this calls for the establishment of validated surveillance-response systems [28]. The First Forum on Surveillance Response System Leading to Tropical Diseases Elimination brought together scientists, diseases control managers and experts from different disciplines and countries to discuss surveillance-response approaches, the most promising experience so far made and the avenues to be pursued in the future. Case studies were discussed in detail such as elimination of malaria in Zanzibar [29,30] and lymphatic filariasis in P.R. China [31]. In Zanzibar, early detection of unusual events is particularly important for effective and timely action, monitoring and evaluation of interventions. It is also critical for the guidance of the selection of appropriate corrective measures to reduce malaria transmission by mobile phone system [30]. In P.R. China, stopping mass chemotherapy against lymphatic filariasis and intensifying the surveillance after the microfilaria rate had fallen to the 1% prevalence level, i.e. under the threshold of transmission, was the critical step in achieving national elimination of this disease [31,32]. The reported experience also trigged renewed efforts to eliminate other NTDs, such as visceral leishmaniasis, onchocerciaisis, and schistosomiasis [1].

Shifting from measuring morbidity and mortality to detecting infections and measuring transmission is a major move transferring the emphasis from general control to elimination. Now surveillance-responses, based on the idea of “surveillance as an intervention tool”, become the key activities [14], leading to a transition stage between control and elimination, which requires the institution of the surveillance-response systems. At this stage, emphasis should be on: (i) standard case definitions to identify and report priority diseases, (ii) collecting and using surveillance data to alert higher levels and trigger local public health action, (iii) investigating and confirming suspected outbreaks or public health events using laboratory confirmation when identified, (iv) analyzing and interpreting data collected in outbreak investigations and data from routine monitoring of other priority diseases, (v) using data analysis to implement an appropriate response, (vi) providing feedback within and across levels of the health system, and (vii) evaluating and improving the performance of the surveillance-response systems [12,19,33]. At the elimination stage, on the other hand, the surveillance-response systems need to focus on the following four aspects: (i) rapid detection of existing, new or re-introduced (e.g., crossing country and regional borders) infections, (ii) identification of areas of low transmission (e.g., from symptomatic and asymptomatic infections), (iii) understanding trends in disease incidence and prevalence (shifts in age groups, increasing parasite heterogeneity, changes in seasonality), and (iv) detection of possible drug resistance [30,34-38].

**Figure 1:**
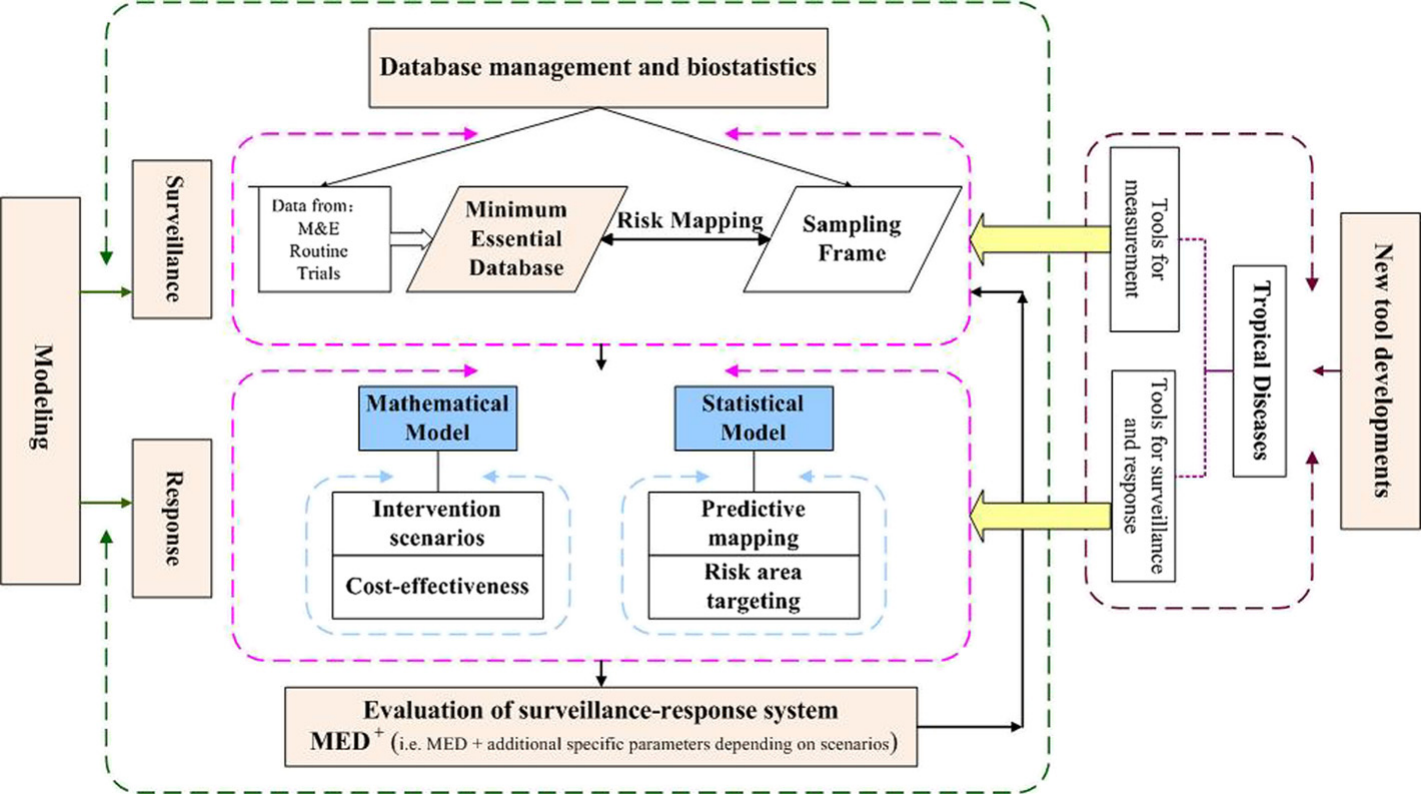
**The major components of a surveillance-response system.** Four components are crucial: (i) a minimum database/dataset for data collection and management, (ii) data modelling by either statistical or mathematical models to forecast the future tendency of disease transmission, thereby providing the guidance for intervention, (iii) novel tools to sensitively detect or respond to low-transmission patterns and (re-)emerging pathogens, and (iv) evaluation of the chosen elimination programme. The overall goal will depend on additional indices to provide the threshold of zero transmission and specific parameters depending on specific scenarios (MED = Minimum Essential Database/set, M&E = Monitoring and Evaluation). (This figure was contributed by Dr. Guo-Jing Yang, and Dr. Jing-Fan Xu).

With challenges in the national elimination programme not only with respect to the disease landscape, but also regarding the broader context of changes with respect to disease foci or outbreak events, we have to make sure that the surveillance-response systems function well. This requires two kinds of action. The first is to establish a good surveillance-response system which consists of at least four components, which include: (i) the definition of the minimum essential data for surveillance, (ii) modelling to forecast/predict disease transmission or possible reintroduction, (iii) develop novel tools to sensitively detect or respond to low-transmission patterns and (re-)emerging pathogens, and (iv) evaluation of the elimination programme that needs additional indices to provide the threshold of zero transmission and specific parameters depending on scenarios (Figure 1).

The second is to promote innovations in the surveillance-response systems. For instance, for malaria to be eliminated, the basic reproduction rate (the number of new malaria cases generated by a single case over the duration of an infection) has to be less than one [39]. With the existing arsenal of tools, only the density of mosquitoes, their daily survival rate, their biting rate and the duration of infection in humans can be manipulated by intervention. Current antimalarial interventions lead to a reduction in the basic rate of malaria reproduction by reducing human infectivity with early and effective treatment and reducing vector capacity by mos-quito control measures [40]. However, when taking into account such changes of environmental and social patterns, such as increased migration to cities with subsequent increase in health conditions, climate change and shifting disease patterns, wider access to wireless technologies (cellular phones and the Internet), innovative surveillance and response are also highly accessible in remote areas where people live under poor conditions [30,41]. The capacity of surveillance and response is fuelled by heightened awareness of the importance of national core capacities for surveillance and response demonstrated by adoption of the International Health Regulations [42].

The First Forum on Surveillance Response System Leading to Tropical Disease Elimination identified six research priorities to strengthen the surveillance-response systems within national elimination programmes:

Dynamic mapping of “pockets” of transmission and/or disease reintroduction;

Dynamic, near real-time capture of population dynamics;

Modelling to optimize surveillance and response systems with regards to the minimum essential data required for surveillance in space and time and for estimating/predicting outcomes and impact of different response packages;

Use of new technologies in elimination strategies supported by mobile and electronic (m- and e-health) –based approaches as well as improved and more sensitive strategies of diagnosis;

Design of response packages tailored to different transmission settings and levels; and

Continuous validation of approaches and response packages.

The First Forum on Surveillance Response System Leading to Tropical Diseases Elimination was held in Shanghai in June 2012 due to the higher awareness of the possibility to move towards elimination of different national diseases. The meeting outlined the research priorities needed and emphasized the development of surveillance-response systems that take into account the One world-One health approach realizing the crucial importance of this approach. Due to the fact that most NTDs are endemic in resource constrained countries, the enormous gaps in the area of surveillance-response systems in the poor countries can only be overcome by a renewed efforts of applied research complemented by a strengthening the health system as well as the development of effective and novel tools tailored to local settings [43]. However, acceptability of new tools for surveillance and response is governed by socio-cultural and political factors, which also need to be taken into account when tailoring the integrated disease control or elimination to a specific setting [44].

## Competing interests

The authors declare that there are no competing interests.

## Authors’ contributions

XNZ, and RB conceived and wrote the first version of the manuscript. XNZ, RB and MT revised the manuscript. XNZ finalized the manuscript. All of authors read and approved the final version of the manuscript.
